# Supplementary material for: A chimeric, half-life extended lysin with a unique mode of action
Source: Front Microbiol. 2026 Aug 21;17:1880134. doi: 10.3389/fmicb.2026.1880134 (PMC13544749; doi:10.3389/fmicb.2026.1880134)
Supplement: Supplementary file 1 [file Data_Sheet_1.DOCX]

Supplementary Material

# 1 Supplementary methods

*PK studies*

In the PK studies, each test article was administered at 17.5 mg/kg once as a slow bolus intravenous (IV) injection via a lateral tail vein in a dose volume of 5 mL/kg to a cohort of 3 mice. Following test article administration, approximately 20 µL of whole blood was collected from each mouse via the lateral tail vein in sampling tubes as per the dose/sampling schedule (*^EC^*LSN_wt_-FLAG: 5 min, 15 min, 30 min, 1h, 2h, 4h, 8h, 24h; Fc-L2-CHAP_opt_-LSN_HEK_: 5min, 15min, 30min, 1h, 2h, 4h, 10h, 24h, 48h, 72h, 96h). At the end of study (24h or 96h) samples were collected via cardiac puncture under terminal isoflurane anesthesia. Plasma was separated by centrifugation at 10,000 x g for 5 min at 4°C. All samples were immediately frozen in liquid nitrogen and stored at -80°C prior to analyses.

*Detection of FLAG-tagged Lysostaphin (FLAG/HIS ELISA)*

Levels of FLAG-tagged Lysostaphin (*^EC^*LSN_wt_-FLAG) in mouse plasma were quantified using the following setup: plates (Sigma Aldrich, M5785-1CS) were coated o/n at 4°C with anti-PreScission antibody (Anti-Human Rhinovirus 3C protease antibody, abcam, ab183574) diluted in coating buffer (Sigma Aldrich, C3041-50CAP). Blocking was done for 1h at RT with SuperBlock Blocking Buffer (Thermo Scientific, 37580). Samples were diluted 1:20 in dilution buffer (DPBS + 0,05% Tween20 [v/v]), and further serially diluted 1:5 in dilution buffer, added to the plates and incubated 1h at RT. Bound analyte was detected using monoclonal anti-FLAG M2-peroxidase (HRP) (Sigma Aldrich, A8592-1MG) and commercial TMB solution (Sigma-Aldrich T0440). A standard dilution series was loaded on each plate in a concentration range 200 - 3.125 ng/mL. Signal from the standard curve was fit in GraphPad PRISM to a sigmoidal curve (4PL) and used to interpolate the lysin concentration in the plasma samples. Area under the curve (AUC) was calculated using GraphPadPrism.

*Detection of intact Fc-CHAP-LSN in mouse plasma*

Levels of Fc-CHAP-LSN in mouse plasma were quantified using a sandwich ELISA approach. In short, plates (Sigma Aldrich, CLS9018-100EA) were coated o/n at 4°C with 100 µL of anti-human IgG (Fc AffiniPure Donkey Anti-Human IgG (H+L), Jackson ImmunoResearch, JAC709005149) diluted in coating buffer (Sigma Aldrich, C3041-50CAP). Plates were washed 3 x with 300 µL DPBS supplemented with 0.05% TWEEN-20 [v/v] (DPBS-T) followed by a blocking step with 200 µL SuperBlock (Thermo Fisher Scientific, 37580) per well for 1 h at room temperature (RT). Afterwards plates were washed again 3x as described above. Plasma samples were diluted 1:100 in SuperBlock, and further serially diluted (5-fold) in 1% mouse plasma (MoP) in SuperBlock. A standard dilution series in 1% MoP in Superblock was prepared in 2-fold dilutions from 256-0.25 ng/mL. 48 µL per well of sample or standard dilution was added to the plates and incubated for 1 h at RT, followed by three washes as described above. Bound analyte was detected using 100 µL of biotinylated anti-L0889 rabbit polyclonal antibody (Genscript, U7045678G0) diluted to 0.004 µg/mL in Superblock and incubated for 1 h at RT, followed again by three washes as described above. 100 µl per well of Poly-HRP streptavidin (Thermo Fisher Scientific, 21140) diluted 1:2500 in Superblock was added to the plates and incubated for 1 h at RT, followed by five washes as described above. After the last washing step, plates were incubated in washing buffer for 5 min at RT. For detection, 100 µL per well of TMB solution (Sigma Aldrich, T0440) was added and incubated for 2-5 min followed by addition of 100 µL per well of 2 N sulphuric acid to stop the reaction. Signal was recorded at 450 nm in a Sunrise plate reader (Tecan). Signal from the standard curve was fit in GraphPad PRISM to a sigmoidal curve (4PL) and used to interpolate the lysin concentration in the plasma samples. Area under the curve (AUC) was calculated using GraphPadPrism.

*Purification of Fc-CHAP-LSN/Fc-CHAP heterodimer*

For the generation of Fc-CHAP-LSN/Fc-CHAP heterodimers, DNA for Fc-CHAP-LSN and Fc-CHAP were co-transfected into Expi293 cells; Fc-CHAP-LSN being N-terminally His-tagged and Fc-CHAP being N-terminally streptavidin tagged. Transfection and subsequent IMAC purification were carried out as described above, yielding a mix of Fc-CHAP-LSN homodimers and Fc-CHAP-LSN/Fc-CHAP heterodimers. Subsequently a Strep-Tactin®XT (IBA) resin was used to purify the heterodimer from the homodimer. 2 mL of slurry were equilibrated with 10 CV volumes of 20 mM HEPES pH7.0, 150 mM NaCl using gravity disposable columns and the elutions from the IMAC purification were added to the resin and incubated on a rotor for 1h at 4°C. The slurry was centrifuged at 1,000 x g for 5 min at 4°C resuspended in 20 mM HEPES pH7.0, 150 mM NaCl and washed with 10 column volumes of 20 mM HEPES pH7.0, 150 mM NaCl. Elution was performed using 5 column volumes of 2.5 mM Desthiobiotin in 20 mM HEPES pH7, 150 mM NaCl. Pooled eluates were supplemented with 1 mM DTT and HRV 3C protease (produced in-house) at a 1:100 protease:substrate molar ratio, followed by dialysis against 300 volumes of formulation buffer (20 mM HEPES (pH 7.0), 150 mM NaCl). Samples were sterile filtered (0.2 µm cutoff filters), aliquoted and frozen at -80 °C for long-term storage. Protein concentrations were determined by UV absorbance using molar extinction coefficients calculated from the amino acid sequence of each lysin variant using the Geneious software.

*Small-scale expression and activity testing in supernatant*

*Transfection and harvest.*

DNA concentrations were normalized to 100 ng/µL. DNA was mixed with transfection reagents, followed by transfer onto 1 mL Expi293 cultures in 24 well format. Transfections, enhancer addition and cell culture conditions were as in the main text. Supernatants were finally harvested (300 × g, 5 minutes), aliquoted and supplementation with 0.5 0M Tris(2-carboxyethyl)phosphine (TCEP). Samples were either processed directly or frozen at -80 °C.

*High throughput growth inhibition assay in supernatants*.

Overnight culture of *Staphylococcus. aureus* ATCC 43300 was grown in tryptic soy broth (TSB) at 37 °C and shaking at 220 rpm, diluted 1:20 into TSB to an optical density at 600 nm (OD_600_) of ~0.05, and further grown until reaching mid-log phase (OD_600_ ~1.0). This culture was then diluted in cation-adjusted Mueller–Hinton broth (caMHB) supplemented with 20% heat-inactivated horse serum to reach a final inoculum of ~5 × 10^5^ CFU/mL. A 2-fold dilution series of the supernatant was prepared in caMHB supplemented 20% heat-inactivated horse serum. Reactions were set up in 96-well U-bottom plates by mixing 30 µL of supernatant solution with 70 µL bacterial suspension. Plates were statically incubated at 37 °C for 17‑19 h. Minimal inhibitory concentration (MIC) was defined as the lowest concentration of lysin that still prevented growth by visual inspection.

*OD reduction assays.*

*S. aureus* BAA-1717 was cultured aerobically on Columbia Agar with 5% sheep blood or in TSB at 37 °C. Pre-cultures were prepared by inoculating TSB with a few bacterial colonies grown on an agar plate, followed by overnight incubation. The pre-culture was used to inoculate 100 mL of TSB at an initial OD_600_ of 0.05. Cells were grown in baffled flasks at 37°C and 130 rpm until an OD_600_ of ~1.1 was reached. The bacterial cells were subsequently harvested by centrifugation (4000 × g, 10 min). Cells were washed twice in DPBS supplemented 1 mM CaCl_2_ and 0.5 mM MgCl_2_, followed by resuspension in the same buffer at a final OD_600_ of 2. Supernatants were diluted 1:8 to 1:16 in Expi293 expression medium supplemented with 0.5 mM TCEP. 100 µL of the diluted supernatants were transferred into a flat-bottomed 96-well plate and mixed with an equal volume of the bacterial suspension, yielding a final OD_600_ of 1. The reaction plate was immediately placed into a Spectramax Plus plate reader (Molecular Devices) pre-heated to 37 °C. OD_600_ was measured at intervals of 20 s over a period of 2 h. Rates of OD reduction were extracted as the maximum slope of the OD reduction curves. To mitigate the impact of noise in the OD data, which can hinder accurate determination of the maximum slope, the data was smoothed using a cubic spline representation. The maximum slope was identified as the minimum of the first derivative of the spline. Data smoothing and slope determination were implemented in Python using the SciPy package and the scipy.interpolate.make_smoothing_spline function with the default lambda parameter.

# 2 Supplementary Figures and Tables

## 2.1 Supplementary Figures

**Supplementary Figure 1:** Graphical representation of the diFc-CHAP-LSN constructs (only one monomer shown). Each construct consists of a signal peptide (SP) combined with purification tags, Fc domain (Fc), a CHAP domain and LSN domain. The SP represents the murine Ig κ light‑chain signal peptide, a TG dipeptide, an N‑terminal hexahistidine tag, and a HRV 3C protease cleavage site. The Fc represents the IgG1 Fc domain. L1 refers to the helical linker with 8 repeats while L2 and L2a refer to the helical linkers with 14 repeats plus a 12 amino acid serum stable peptide. L2 and L2a only differ in the composition of the 12 amino acid peptide. CHAP refers to the CHAP domain of L2-1, CHAP_opt_ is the CHAP domain of L2-1 with an additional Y46D mutation and CHAP_opt_var_ contains an additional K143A mutation. Inactive variants are marked with an asterisk (CHAP: C27S; LSN: H82A or H113A). Display constructs contain a C-terminal PDGFR transmembrane domain.

**Supplementary Figure 2:** Graphical representation of the homodimer diFc-CHAP-LSN, homodimer diFc-CHAP and the heterodimer Fc-CHAP-LSN:Fc-CHAP. Each construct consists of a signal peptide (SP) combined with purification tags, Fc domain (Fc), a CHAP domain and LSN domain. The SP represents the murine Ig κ light‑chain signal peptide, a TG dipeptide, an N‑terminal hexahistidine tag, and a HRV 3C protease cleavage site. The Fc represents the IgG1 Fc domain. L1 refers to the helical linker with 8 repeats. CHAP refers to the CHAP domain of L2-1.

**Supplementary Figure 3:** PK profiles of *^EC^*LSN_wt_-FLAG and Fc-L2-CHAP_opt_-LSN_HEK_. Purified lysins were injected as a single slow bolus intravenous injection via a lateral tail vein into CD1 mice at a dose of 17.5 mg/kg. Blood samples were drawn at indicated timepoints and lysin levels quantified by ELISA. Points on the graph represent a mean measured blood concentration of n=3 with standard deviation. Note that we show a composite of two separate experiments, with *^EC^*LSN_wt_-FLAG (experiment 1; last measured timepoint at 24 h) having been measured separately to Fc-L2-CHAP_opt_-LSN_HEK_ (experiment 2; last measured timepoint at 96 h). LLOQ was 0.05 µg/mL. Area under the curve (AUC in µg/mL*h) was 8-9 fold higher for the half life extended lysin compared the non-Fc bearing lysin.

**Supplementary Figure 4:** Western blot analysis of PK samples for Fc-L2-CHAP_opt_-LSN_HEK_ under reducing conditions. Left lanes 1-5 show standards diluted in 1.15% CD1 mouse plasma (load on gel from 54 ng to 3.375 ng in 2-fold dilutions). Right: Plasma samples taken at designated timepoints (in min) diluted to contain 1.15% mouse plasma; P: 1.15% CD1 mouse plasma; x: empty well; M: molecular weight marker. Detection with a rabbit anti-lysostaphin antibody generated in house. Exposure time 5 min. For the time points shown, no cleaved lysostaphin could be detected in these uninfected mice. Analysis of later timepoints was not possible due to the detection limit of the assay.

**Supplementary Figure 5:** Western blot analysis of diFc-CHAP-LSN variants in supernatant. (A) Western blot of a time-course secretion experiment in which Fc-L1-CHAP-LSN_HEK_ is compared to the Fc-L1-CHAP*-LSN_HEK_, Fc-L1-CHAP-LSN*_HEK_ and Fc-L1-CHAP*-LSN*_HEK_. Aliquots were taken after 6, 24, 48 and 72 hours. Where the active variant results in the appearance of three fragmentation bands which correspond to the release of LSN (27 kDa) and two CHAP-LSN fragments (33 and 37 kDa). A faint LSN band can be observed in the Fc-L1-CHAP*-LSN_HEK_ sample around 27 kDa. For all constructs faint fragmentations bands can be observed at 40 and 57 kDa, assumed to be independent of the catalytic activity of the constructs. The original uncropped western blot is shown in Supplementary Figure 10A. (B) Western blot of the supernatant samples in which single glycine was substituted within the GGG stretches of Fc-L2a-CHAP_opt_-LSN_HEK_ to alanine. Six CHAP variants (CHAP_opt-G85A,_ CHAP_opt-G86A,_ CHAP_opt-G87A,_ CHAP_opt-G115A,_ CHAP_opt-G116A_ or CHAP_opt-G117A_) and three LSN variants (LSN_HEK-G62A_, LSN_HEK-G63A_ or LSN_HEK-G64A_) were generated. The western blot was overloaded to reveal the presence of truncation products. Four lower molecular truncation products can be observed (27 – 33 – 37 – 40 kDa). Point mutations in the CHAP_opt-85GGG87_ stretch result in the loss of signal of the 37 kDa band, while the point mutations in the CHAP_opt-115GGG117_ stretch result in the loss of signal of the 33 kDa band. The mutations in LSN result in severely reducing the activity of the constructs, less to no degradation bands are observed at 27 kDa, 33 kDa or 37 kDa. The closest related reference sample (Fc-L2-CHAP_opt_-LSN_HEK_) on the same gel is shown as a reference. The original uncropped western blot is shown in Supplementary Figure 10B. (C) Secretion yield and activity data of the mentioned constructs in supernatant. Secretion yields were quantified by SDS-PAGE, OD reduction assay rate (ORA rate) was performed with *S. aureus* BAA-1717 and the MIC was determined for *S. aureus* ATCC 43300.

.

**Supplemental Figure 6:** Expression and stability profile of Fc-L1-CHAP_opt_-LSN_HEK_. (A) SDS–PAGE analysis of purified Fc-L1-CHAP_opt_-LSN_HEK_ under reducing conditions. The protein sample (2.5 µg) were resolved on an AnykD Criterion™ TGX precast gel and visualized by InstantBlue® protein stain. Molecular weight markers (kDa) are indicated. A predominant band at the expected molecular weight is observed, consistent with high sample purity. (B) LSN-induced LSN-release from Fc-L1-CHAP_opt_-LSN_HEK_. Western blot corresponding with the data from Figure 4. Purified LSN_HEKa_ was incubated at increasing concentrations in 80% human serum and in presence of 10^8^ CFU/mL *S. aureus* ATCC 43300 with or without a fixed concentration of Fc-L1-CHAP_opt_-LSN_HEK_ (1 µM) for up to 3h at 37°C. Samples were analyzed by SDS-PAGE followed by western Blotting to quantify the amounts of free LSN in each reaction.

**Supplementary Figure 7:** Uncropped western blots used for quantification in Figure 5**.** Purified Fc-L1-CHAP_opt_-LSN_HEK_ (0.5 µM) was incubated in presence of DPBS (top) or 80% human serum (bottom) with increasing CFU/mL of *S. aureus* BAA-1717 at 37°C. Samples of each condition were taken after 3, 5 or 24 hours and were analyzed by SDS-PAGE, followed by Western blotting using a LSN-directed antibody for detection. If no bacteria were added the lane was denoted with “-“. STD: standard Fc-L1-CHAP_opt_-LSN_HEK_ at 0.5 µM. CTRL: Control sample with bacteria only at different CFU/mL. Boxes highlight the area of the blot depicted in Figure 5A.

**Supplementary Figure 8:** Mass spectrometry–based mapping of the cleavage site in variant Fc-L2a-CHAP_opt_var_-LSN_HEK_. (A) Amino acid sequence of Fc-L2a-CHAP_opt_var_-LSN_HEK_ shown with residue numbering. The Fc region is shown in blue, the helical linker in black, CHAP in grey, PepM23 in light green, SH3 domains in light green. The proposed cleavage site GG is indicated in red. (B) Representative SDS-PAGE analysis of purified Fc-L2a-CHAP_opt_var_-LSN_HEK_ showing the full-length protein and a stable ~50 kDa cleavage product. Schematic cartoons illustrate the domain architecture of the full-length construct and the truncated species. (C) Peptide abundance profile along the Fc-L2a-CHAP_opt_var_-LSN_HEK_ sequence derived from FragPipe analysis of the 50 kDa gel slice. Following tryptic digestion and LC–MS/MS, peptide peak areas were extracted using FragPipe (24.0) and plotted from the N to the C-terminus. Only fully tryptic peptides without missed cleavages were included. Peptides C-terminal to Lys464 exhibit strongly reduced signal intensities relative to upstream peptides, despite the predominance of full-length protein in the sample, consistent with a C-terminal truncation occurring at the CHAP–LSN junction. (D) Intact mass analysis of PNGase F treated Fc-L2a-CHAP_opt_var_-LSN_HEK_ showing a dominant molecular species with a mass shift of approximately +43 Da relative to the theoretical full-length sequence mass and a distinct truncated species (50933.7 Da). (E) Intact mass analysis of PNGase F treated Fc-L2-CHAP_opt_-LSN_HEK_ showing the non-oxidized full-length form as the main peak, accompanied by satellite peaks with mass shifts of approximately +42 to +48 Da.

**Supplementary Figure 9:** Uncropped western blots from figures in the main text. (A) Uncropped Western blot for Figure 3; the boxed region denotes the cropped area presented in the main figure. (B) Uncropped Western blot for Figure 6A; the boxed region denotes the cropped area presented in the main figure. (C) Uncropped Western blot for Figure 7A; the boxed region denotes the cropped area presented in the main figure.

**Supplementary Figure 10:** Uncropped western blots from figures in the supplementary information. (A) Full‑length Western blot for Supplementary Figure 5A; the boxed region denotes the cropped area presented in the main figure. (B) Full‑length Western blot for Supplementary Figure 5B; the boxed regions denote the cropped areas presented in the main figure.

**Supplementary Table 1:** Thermal stability and production yield of lysin constructs in three different formulation buffers.
Thermal unfolding temperatures (Tm) and production yields are reported as mean ± SD across independent production batches for each lysin construct. Data are grouped by formulation buffer as follows: MES buffer (50 mM MES, pH 6.5, 300 mM NaCl, 1 mM CaCl₂, 5% glycerol, 50 mM arginine, 2 mM DTT), Histidine buffer (100 mM L‑histidine, pH 6.5, 300 mM NaCl, 1 mM CaCl_₂_, 5% glycerol), and HEPES buffer (20 mM HEPES, 150 mM NaCl, pH 7.0).

| **MES buffer** | | | | | | |
| --- | --- | --- | --- | --- | --- | --- |
| **Lysin** | **n (Tm)** | **Tm mean (°C)** | **Tm SD (°C)** | **n (Final yield)** | **Final yield mean (µg/mL)** | **Final yield SD (µg/mL)** |
| Fc-L1-CHAP-LSN_HEK_ | 5 | 52.5 | 3.2 | 7 | 13.0 | 9.8 |
| Fc-L1-CHAP_opt_-LSN_HEK_ | 1 | 53.1 | NA | 3 | 66.0 | 35.8 |
| Fc-L2-CHAP_opt_-LSN_HEK_ | 1 | 54.6 | NA | 1 | 84.0 | NA |
| Fc-L2a-CHAP_opt_-LSN_HEK_ | 1 | 55.8 | NA | 1 | 34.4 | NA |
| **Histidine buffer** | | | | | | |
| **Lysin** | **n (Tm)** | **Tm mean (°C)** | **Tm SD (°C)** | **n (Final yield)** | **Final yield mean (µg/mL)** | **Final yield SD (µg/mL)** |
| Fc-L1-CHAP_opt_-LSN_HEK_ | 4 | 52.0 | 3.1 | 2 | 28.2 | 24.1 |
| Fc-L2-CHAP_opt_-LSN_HEK_ | 2 | 53.4 | 0.5 | 1 | 14.8 | NA |
| Fc-L2a-CHAP_opt_-LSN_HEK_ | 1 | 53.6 | NA | 0 | NA | NA |
| **HEPES buffer** | | | | | | |
| **Lysin** | **n (Tm)** | **Tm mean (°C)** | **Tm SD (°C)** | **n (Final yield)** | **Final yield mean (µg/mL)** | **Final yield SD (µg/mL)** |
| Fc-L1-CHAP-LSN_HEK_ | 1 | 45.1 | NA | 6 | 9.5 | 6.8 |
| Fc-L1-CHAP*-LSN_HEK_ | 1 | 57.7 | NA | 1 | 61.0 | NA |
| Fc-L1-CHAP_opt_-LSN_HEKa_ | 1 | 50.6 | NA | 4 | 48.4 | 16.4 |
| Fc-L1-CHAP-LSN*_HEK_ | 1 | 55.1 | NA | 1 | 11.7 | NA |
